# Epigenomic and genomic analysis of transcriptome modulation in skin cutaneous melanoma

**DOI:** 10.18632/aging.103115

**Published:** 2020-07-07

**Authors:** Wuzhen Chen, Pu Cheng, Jingxin Jiang, Yunqing Ren, Dang Wu, Dan Xue

**Affiliations:** 1 Department of Surgical Oncology, The Second Affiliated Hospital of Zhejiang University School of Medicine, Hangzhou Zhejiang, P.R. China; 2 Department of Gynecology, The Second Affiliated Hospital of Zhejiang University School of Medicine, Hangzhou Zhejiang, P.R. China; 3 Department of Dermatology, The Second Affiliated Hospital of Zhejiang University School of Medicine, Hangzhou Zhejiang, P.R. China; 4 Department of Radiation Oncology, The Second Affiliated Hospital of Zhejiang University School of Medicine, Hangzhou Zhejiang, P.R. China; 5 Department of Plastic Surgery, The Second Affiliated Hospital of Zhejiang University School of Medicine, Hangzhou Zhejiang, P.R. China; 6 Key Laboratory of Tumor Microenvironment and Immune Therapy of Zhejiang Province, Hangzhou, China

**Keywords:** skin cutaneous melanoma, genomic analysis, epigenomic analysis, transcriptome modulation, multi-omic analysis

## Abstract

Skin cutaneous melanoma (SKCM) is characterized by both epigenetic DNA methylation (MET) abnormalities and genomic copy number variations (CNVs). The resulting transcriptome dysregulation promotes progression of many cancers. In this study, DNA copy numbers and MET, as well as mRNA expression, were examined in 466 SKCM samples from The Cancer Genome Atlas. Our results indicate that CNVs-correlated (CNVcor) genes and MET-correlated (METcor) genes are coregulated to a remarkable degree. In addition, integrative multi-omics analysis of both METcor and CNVcor genes revealed four SKCM subtypes with differing prognoses; these subtypes were validated with independent data. Immune cell scores were markedly elevated in the iC1 subtype, which had the best prognosis. Immune cell infiltration correlated with DNA MET or CNV level in SKCM. In the iC3 subtype, which was associated with the most aggressive SKCM cases, FAM135B gene mutation frequencies were increased, while CD8A, GBP5, KIAA0040, and SAMHD1 expression were downregulated, suggesting that these genes play important roles in cancer development and immune responses. Taken together, the results of our epigenetic and genomic transcriptome modulation analysis improve our understanding of SKCM pathobiology and may aid in the development of more effective therapies.

## INTRODUCTION

Incidence of skin cutaneous melanoma (SKCM), a common malignant skin tumor, has increased rapidly over the past decade [1]. In the USA, an estimated 87,000 new melanoma cases and 9,000 melanoma-related deaths occurred in 2017 [2]. Although novel treatments might help improve clinical outcomes, the prognosis for this disease remains poor due at least in part to the involvement and thickness of the lymph nodes and the ability of malignant cells to colonize distant organs [3, 4]. A comprehensive understanding of the molecular features of melanoma will help improve treatment efficacy.

Recently, large-scale multi-omics studies have greatly increased our understanding of genomic and epigenetic dysregulation in many diseases [5]. Genome alternations, like DNA mutations or copy number variations (CNVs), frequently take place during tumorigenesis and can promote cancer development [6]. Although DNA mutations have been used to guide SKCM subtyping and prognosis prediction, recent advances in SKCM treatment have mainly come from immunotherapy. It is therefore necessary to investigate genomic and epigenomic abnormalities in SKCM from a novel perspective. In addition, epigenetic regulation in the form of DNA methylation (MET) contributes to many SKCM characteristics [7, 8]. DNA CNVs are important regulators of SKCM development, and resulting transcriptional dysregulation might drive the progression of SKCM [9, 10]. In addition, DNA MET profiling studies indicate that epigenetic regulation influences biological and clinical aspects of cancer development [11, 12]. Some crucial cancer-related genes, such as LKB1, RB1, and RASSF1A, can regulate DNA MET, thus modulating gene function [13–15].

Correlations between CNVs and MET have been found in multiple studies, which suggest that DNA MET is both trans-regulated by DNA CNVs and related to the redistribution of methylase complexes [16–19]. However, the epigenetic relationship between DNA CNVs and MET in SKCM progression remains unclear. In this study, we investigated regulatory relationships between DNA MET and CNVs in SKCM and whether they are associated with prognosis. DNA copy numbers and MET, as well as messenger RNA (mRNA) expression, were examined in SKCM samples. Genes for which expression was correlated with DNA MET (METcor) or copy number (CNVcor) were then identified. In addition, multi-omics integration analysis of METcor and CNVcor genes was conducted to identify molecular subtypes associated with differences in SKCM prognosis. Finally, deeper systematic analysis was used to identify novel biomarkers and targets for distinguishing cancer subtypes.

## RESULTS

### DNA MET and copy number dysregulation at the transcriptomic level

MET, CNV, and mRNA expression profiles were obtained from 466 SKCM samples in TCGA. Original data were then preprocessed as described in Materials and Methods. Correlation coefficients between DNA MET or CNV profiles and mRNA expression profiles were calculated to assess the effects of epigenomic and/or genomic aberrations. Correlation coefficient r values were normalized using Fisher’s Z-transformation to stabilize variance.

**Figure 1:**
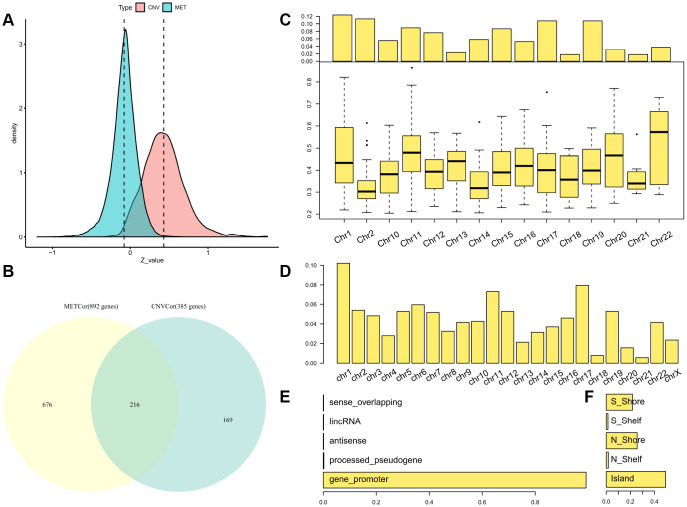
**Identification of DNA copy-number-correlated (CNVcor) and DNA methylation-correlated (METcor) genes in SKCM.** (**A**) Distribution of correlation coefficients between mRNA expression levels and DNA copy number or DNA methylation across samples. (**B**) Venn diagram showing counts of CNVcor genes, METcor genes, and overlapping genes. (**C**) Proportional frequencies of CNVcor genes against total gene counts in each chromosome arm. (**D**) Proportional frequencies of METcor genes against total gene counts in each chromosome arm. (**E**, **F**) Genomic positions of DNA methylation probes are categorized based on positional relations with CpG islands (right) and genes (left).

Consistent with our previous findings, overall correlation coefficients between DNA CNVs and expression profiles showed a marked right-sided skew (skewness = 0.67425, p < 1e^-5^). By contrast, correlation coefficients between DNA MET patterns and expression profiles displayed a left-sided skew (skewness = -0.4274, p < 1e^-5^) (Figure 1A), indicating that abnormalities in DNA MET and CNVs negatively and positively regulated transcription, respectively.

The METcor and CNVcor gene sets included many individual genes (Supplementary Tables 2–3); only genes with notable correlations with overall survival (OS) were included in subsequent analysis (log rank p < 0.05). Positively-correlated gene signatures were typically included in the DNA copy number gene set (CNVcor, n = 385), while negatively-correlated gene signatures were typically included in the DNA MET gene set (METcor, n = 892). CNVcor genes were associated with CNV-dependent dysregulation at the transcriptional level, whereas METcor genes were associated with MET-dependent transcriptional dysregulation at the epigenetic level. In addition to genes that belonged to either the METcor or the CNVcor set, 216 overlapping genes were included in both sets, implying that both METcor and CNVcor genes were specifically associated with dysregulation at the transcription level (Figure 1B).

As was found in a previous study, chromosome 1 was rich in CNVcor genes (Figure 1C), suggestive of regional sensitivity of gene expression to DNA dosage [20, 21]. METcor genes were also preferentially located in certain chromosomal regions, such as chromosomes 17 and 1 (Figure 1D and Supplementary Figure 1); in addition, most METcor genes were protein coding genes (MET on the promoter region) (Figure 1E) and located primarily within CpG islands (Figure 1F). These results indicate that METcor and CNVcor genes were important contributors to transcriptional dysregulation in SKCM.

### Molecular subtypes based on METcor and CNVcor genes

**Figure 2:**
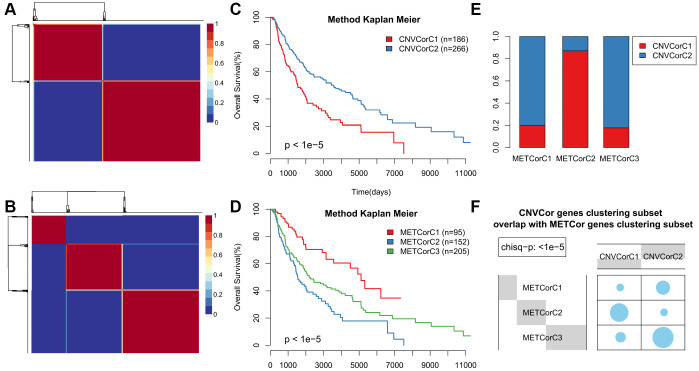
**Identification of SKCM molecular subtypes using CNVcor and METcor genes.** (**A**, **B**) Plots show the non-negative factorization (NMF) cluster results for CNVcor genes in CNV data (**A**) and for METcor genes in MET data (**B**). (**C**, **D**) Kaplan–Meier plot analyses of differences in OS among subtypes identified by NMF clustering of CNVcor (**C**) and METcor (**D**) genes. (**E**, **F**) Subtypes based on CNVcor genes overlapped to a great extent with those based on METcor genes.

Next, we examined whether METcor and CNVcor gene expression could predict prognostic subgroups. Each gene set profile was examined using NMF clustering analysis, with the cluster number k set at 2-10; k values were then determined for all profiles (k=3 for MET and k = 2 for CNV) (Figure 2A, 2B and Supplementary Figures 2–5). Surprisingly, subtype identifications based on CNVcor genes overlapped to a large extent with those based on METcor genes (p < 1e^-5^, χ^2^-test), suggesting that METcor and CNVcor genes both contribute to regulation of SKCM (Figure 2E, 2F). Moreover, Kaplan-Meier (KM) curve analysis indicated that subtypes identified based on either METcor or CNVcor genes were associated with patient OS (Figure 2C, 2D, p < 0.05).

**Figure 3:**
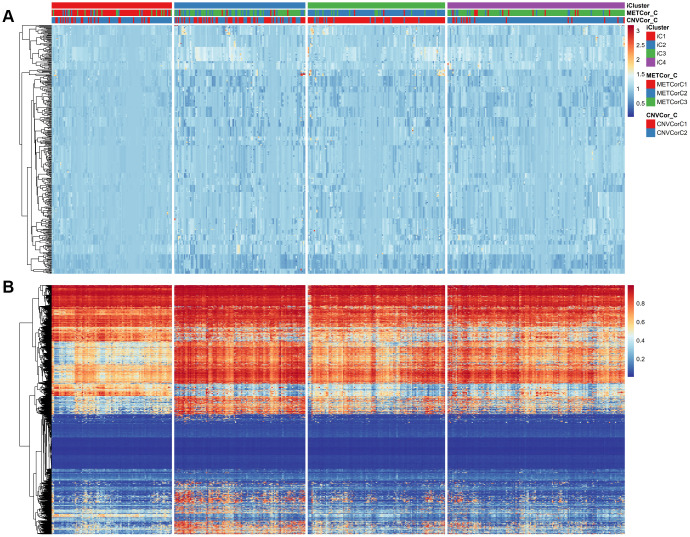
**Expression patterns of subtypes based on CNVcor and METcor genes.** Subtypes based on CNVcor (**A**) or METcor (**B**) genes using NMF cluster methods are indicated with colored bars.

The comprehensive clustering method iCluster was adopted to integrate genomic information regarding DNA MET and CNVs as well as mRNA expression. Clustering analysis was performed using a cluster number k of 2/3. Twenty iterations of clustering analysis at K=2 (category 3) and at K=3 (category 4) were performed to generate optimal iCluster clustering results. Our results indicated that K=3 generated more stable clustering results than K=2 (Supplementary Figures 6, 7). Samples were therefore clustered into four subclasses: iC1-iC4 (n=99, 108, 113, and 146, respectively). Clustering results for these four subclasses are displayed in Figure 3A, 3B; clustering results for each sample are presented in Supplementary Table 4.

**Figure 4:**
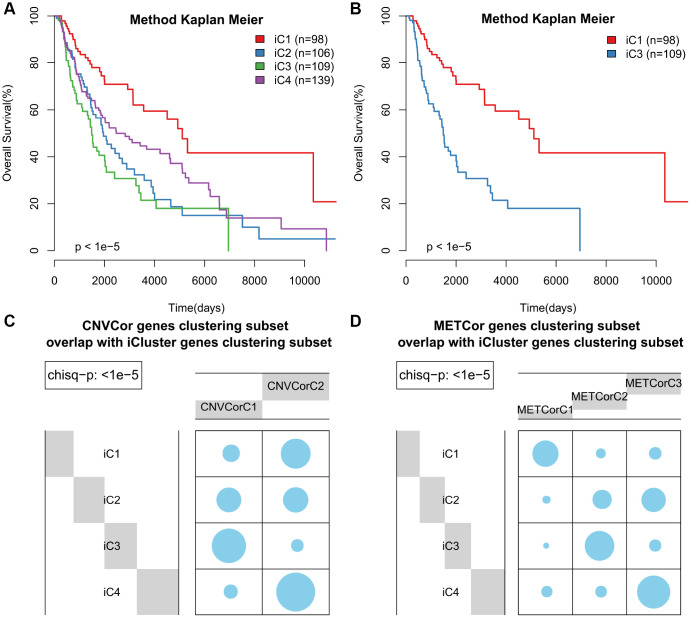
**Identification of SKCM molecular subtypes using iCluster analysis.** (**A**) Kaplan–Meier plot analyses of differences in OS among subtypes identified by iCluster (iC1, iC2, iC3, and iC4). (**B**) Kaplan–Meier plot analyses of OS in iC3 and iC1 subtypes. (**C**, **D**) The subtypes determined through iCluster analysis overlapped extensively with those based on CNVcor (**C**) or METcor (**D**) genes.

According to KM curve analysis, OS was poorest in the iC3 subtype (p < 0.05, Figure 4A). Comparisons of patient OS among the four subgroups (Figure 4B and Supplementary Figure 8) indicated that the iC1 and iC3 subgroups differed most in terms of prognosis (p < 0.001). In addition, the subtypes identified using iCluster largely overlapped with those identified based on METcor and CNVcor genes (p < 1e-5, χ^2^-test, Figure 4C, 4D). These results suggest that integrated analysis of METcor and CNVcor genes identified clinically-relevant molecular subtypes in which epigenomic and genomic transcriptional dysregulation influences prognosis.

### Abnormalities in DNA MET and CNVs are linked

**Figure 5:**
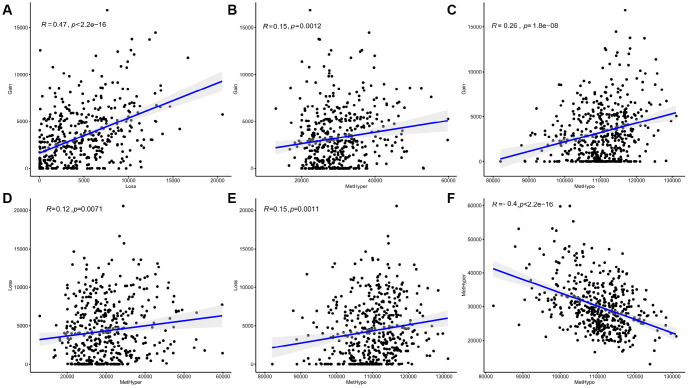
**Abnormal DNA copy numbers and DNA methylation are linked in SKCM.** (**A**, **F**) Abnormalities in DNA copy numbers or DNA methylation were determined based on a cutoff fold difference >0.2 compared to average values in non-tumor tissues. DNA copy-number gains (CNVgain) and losses (CNVloss) and DNA hypermethylation (METhyper) and hypomethylation (METhypo) are shown for each sample. (**B**–**E**) Plots show the pairwise frequencies of CNVgain, CNVloss, METhyper, and METhypo genes in individual samples.

After correcting for batch effects, occurrence rates of genome-wide abnormalities in DNA MET and CNVs were compared. DNA copy-number loss (CNVloss, β<-0.3) and gain (CNVgain, β>0.3), as well as DNA hypomethylation (METhypo, β<0.2) and hypermethylation (METhyper, β>0.8), were also identified based on the predetermined 0.3-fold change threshold and were compared with the average values of each probe in normal tissue. Our results (Supplementary Table 5) indicate that CNVgain and CNVloss frequencies were strongly correlated (p < 1e-5, Figure 5A). In addition, METhyper and METhypo frequencies were also correlated (p < 1e-5, Figure 5F). Abnormalities in directional CNVloss, CNVgain, METhypo, and METhyper were tightly correlated, indicating that each correlation was independent of directional abnormality (Figure 5B–5E, p < 0.001). In summary, our findings indicated that SKCM cases with higher DNA CNV frequencies also tended to have higher frequencies of abnormal DNA MET.

### Identification of key molecular features of SKCM subtypes

**Figure 6:**
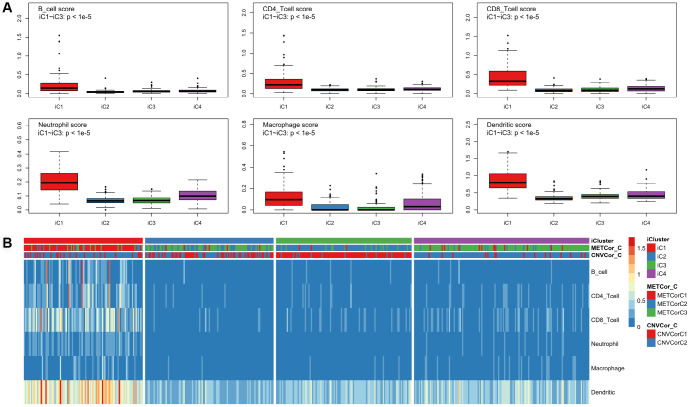
**Identification of key immune features for SKCM subtypes.** Immune scores were calculated for samples from the four subtypes and were compared using the TIMER (tumor immune estimation resource) approach. Scores were determined for six immunocytes in each sample (**A**); these scores were compared among subtypes (**B**).

Differences in clinical characteristics (such as Stage, TNM, Primary Site, and Gender) were examined among the four subtypes identified above. There were no statistically significant differences in clinical characteristics among the four SKCM subtypes (Supplementary Figure 9). In addition, the tumor immune estimation resource (TIMER) approach was used to analyze differences in samples belonging to the four subtypes (Supplementary Table 6) [22]. Six immune cell scores for samples in the iC1 subtype that had the best prognosis were markedly elevated compared to scores for samples in other subtypes (Figure 6A, 6B, p < 0.01). These results suggest that degree of immune cell infiltration or immune microenvironment in SKCM are correlated with DNA MET or CNV levels.

**Figure 7:**
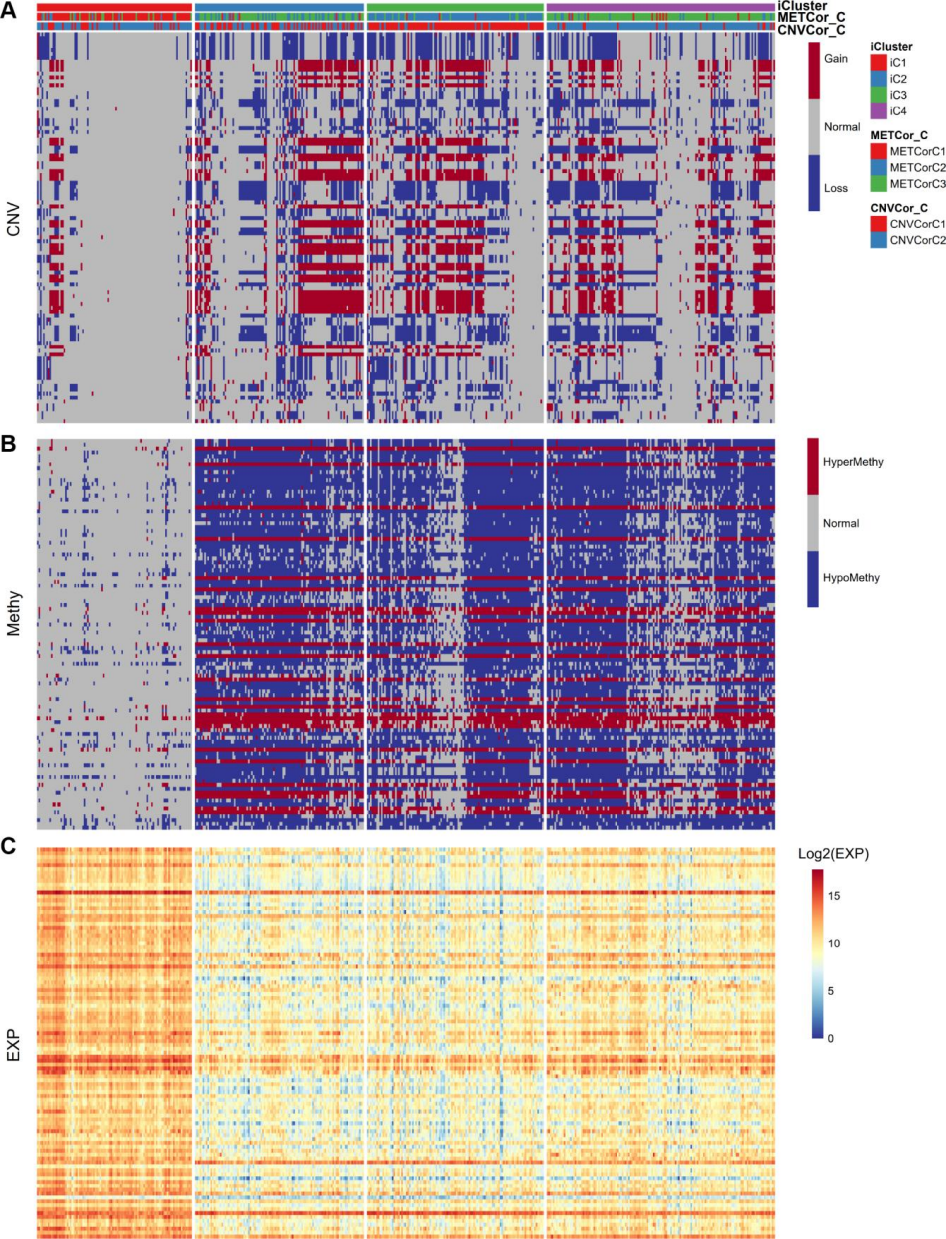
**Identification of general molecular features of SKCM subtypes.** (**A**) Distribution of DNA CNVs in iCluster subtypes. (**B**) Distribution of DNA MET in iCluster subtypes. (**C**) Heatmap of DEGs among iCluster subtypes.

**Figure 8:**
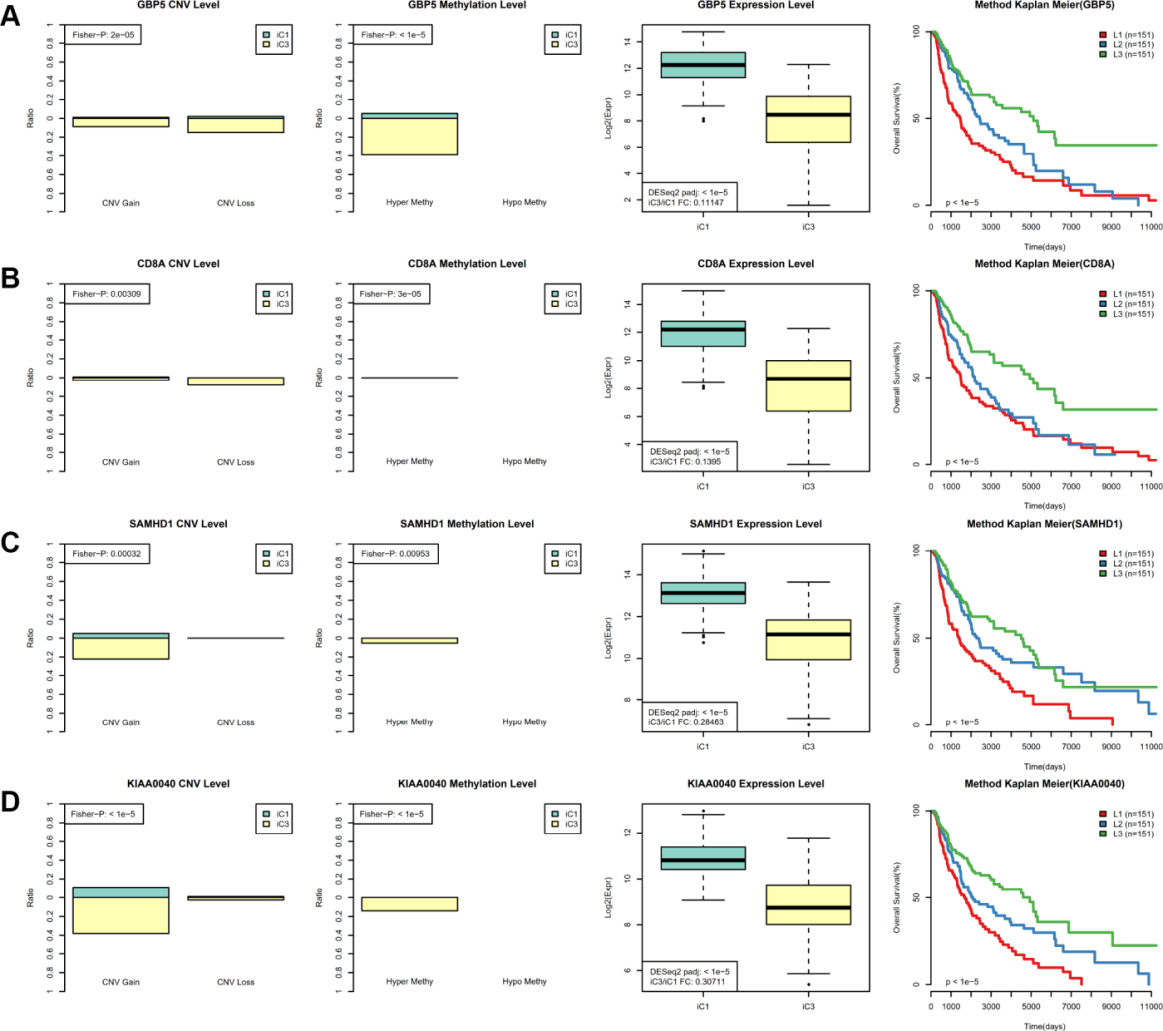
**Identification of key molecular features in SKCM subtypes.** (**A**) GBP5 MET, CNV, and expression levels in the iC1 and iC3 subtypes are shown in the left panel. Samples were divided among high (L3), medium (L2), and low (L1) groups based on GBP5 gene expression; Kaplan–Meier plot analysis for these groups is shown in the right panel. (**B**) CD8A MET, CNV, and expression levels in the iC1 and iC3 subtypes are shown in the left panel; Kaplan–Meier plot analysis for L1, L2, and L3 CD8A groups is shown in the right panel. (**C**) SAMHD1 MET, CNV, and expression levels in the iC3 and iC4 subtypes are shown in the left panel; Kaplan–Meier plot analysis for L1, L2, and L3 SAMHD1 groups is shown in the right panel. (**D**) KIAA0040 MET, CNV, and expression levels in the iC3 and iC4 subtypes are shown in the left panel; Kaplan–Meier plot analysis for L1, L2, and L3 KIAA0040 groups is shown in the right panel.

Next, we examined heterogeneity in DNA MET, CNVs, and gene expression among iC3 and iC1 subtype samples. Three categories each were created for DNA CNV and MET levels: Normal, Loss, and Gain for CNV and Normal, HypoMethy, and HyperMethy for MET. Fisher’s exact test was used to identify DNA MET or CNV genes with levels that differed between the iC3 and iC1 subtypes [23]; the results are shown in Supplementary Tables 7, 8. Differentially expressed genes (DEGs) between the iC3 and iC1 subtypes were identified using DESeq2 (Supplementary Table 9, p < 0.05) [24]. MET, CNV, and expression results for genes with marked differences among samples from the four subtypes are shown in Figure 7. A total of 221 genes with different MET, CNV, and expression levels between the iC3 and iC1 subtypes were examined in univariate survival analysis to identify differences in prognostic characteristics among the subtypes. The results indicated that 146 of these genes were correlated with prognosis (Supplementary Table 10, log rank p < 0.05). Among them, increased hypermethylation and decreased expression of the GBP5, CD8A, SAMHD1, and KIAA0040 genes were associated with poorer outcomes in the iC3 subtype compared to the iC1 subtype. Samples were then assigned to low (L1), medium (L2), or high (L3) groups based on the expression of KIAA0040, SAMHD1, CD8A, and GBP5. The results indicated that prognostic outcomes were positively correlated with the expression of CD8A, GBP5, KIAA0040, and SAMHD1 (Figure 8A–8D), suggesting that CD8A, GBP5, KIAA0040, and SAMHD1 expression levels were associated with DNA CNV or MET level. Next, associations between the abovementioned 146 genes and patient prognosis were evaluated in the GEO GSE65904 SKCM dataset (Supplementary Table 10). Seventy-eight genes were correlated with prognosis in that dataset; survival curves for the top 20 genes are shown in Supplementary Table 11 and Supplementary Figure 10.

**Figure 9:**
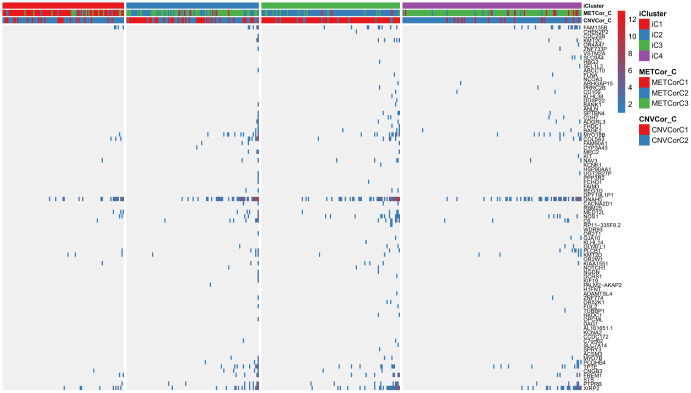
**Differentially mutated genes among the SKCM subtypes.**

Finally, SKCM mutation profiling data were examined to explore associations with the subclassifications identified in this study. Synonymous mutations were removed, and both nonsense and missense gene mutations were included. Overall, mutation frequencies differed significantly among the various subtypes. A selection of 85 genes with mutation frequencies that differed markedly (P < 0.01) between the iC1 and iC3 subtypes based on Fisher’s test is shown in Figure 9; mutation frequency data are shown in Supplementary Table 12, and Fisher’s test results are shown in Supplementary Table 13. For example FAM135B gene mutation frequency was much higher in the iC3 subtype than in the iC1 subtype (p < 0.01). Interestingly, FAM135B gene mutation frequency was also increased in the iC2 and iC4 subtypes, which had poor prognoses, compared to the iC1 subtype (p < 0.05). In summary, these results suggest that DNA copy number- and MET-related molecular subtypes of SKCM are associated with differences in FAM135B gene mutation frequency, which might regulate progression of SKCM.

## DISCUSSION

According to a previous report, integrative analysis of various cancer genome characteristics can identify meaningful molecular subtypes that correspond to mechanistic and clinical heterogeneities in tumors as well as potential biomarkers and treatment targets. However, the complexity of genomic data in cancer remains a challenge. Here, epigenomic and genomic dysregulations of METcor and CNVcor genes were identified based on the TCGA database in an attempt to integrate epigenomic and genomic data in SKCM subtyping. Our findings demonstrated that these correlation genes successfully identified SKCM subtypes that reflected distinct molecular and immunological features and were associated with different outcomes. In addition, SKCM samples with higher frequencies of abnormal CNVcor genes also had higher frequencies of abnormal METcor genes, indicating that patients with higher frequencies of DNA CNVs also had higher frequencies of abnormal DNA MET. These findings suggest that linkages between abnormal DNA MET and CNVs should be considered when examining their effects in SKCM.

Correlations between DNA CNVs and MET have attracted attention in recent years. If CpG methylation were exclusively cis-regulated, it would be controlled only by the surrounding DNA sequence of the same allele and methylation level would remain unchanged regardless of copy number changes. However, several studies have identified correlations between DNA CNVs and MET, suggesting that DNA MET may be trans-regulated by DNA CNVs. Additionally, when copy number increases were present, DNA MET decreased around CpG islands and increased in CpG oceans [16], perhaps due to redistribution of DNA MET from CpG oceans to nearby CpG islands. Oxidative damage has been reported to induce such DNA MET redistribution [25]. Decreased CpG island methylation may result from redistribution of the methylase complex [16, 17], and the magnitude of aging-associated methylation changes is similar to the CNV-MET associations we have observed in tumor samples [18, 19]. In this study, we identified CNVCor and METCor gene subsets based on expression profiles from TCGA-SKCM data. By integrating and clustering the DNA CNV, MET, and mRNA expression multi-omics data, we were able to divide 466 TCGA-SKCM samples among four prognostic subtypes that were validated by independent data. Survival outcomes were significantly better in the iC1 subtype compared to the other subtypes, and subsequent candidate key feature gene analysis was performed to identify genes associated with this difference. Additional studies should be conducted to examine the synergistic relationship between DNA MET and CNVs identified in our retrospective study.

Our classification analysis based on METcor and CNVcor genes might help identify novel precision biomarkers and treatment targets for SKCM. Analysis of different mutation types among SKCM subtypes revealed a difference in frequencies of FAM135B mutation. Specifically, FAM135B mutation frequency, which is generally high among SKCM patients, was lowest in the iC1 subtype, which was associated with the best prognosis. Mutation frequencies for FAM135B, which is associated with cellular lipid metabolic processes, are high in many different malignancies (including esophageal squamous cell cancer and small cell lung cancer) [26, 27]. However, the mechanism by which FAM135B modulates cancer development and progression remains unclear. Moreover, the relationship between FAM135B mutation frequency and its expression in SKCM remains unclear. Our present findings suggest that FAM135B mutation might promote invasive behaviors in iC2-iC4 subtype tumors to some extent, but additional molecular biology experiments and protein-protein (PPI) analyses are needed to verify these results.

We also identified CD8A, GBP5, KIAA0040, and SAMHD1 as potentially crucial regulators of SKCM initiation and progression. GBP5, a member of the guanylate-binding protein (GBP) family, belongs to the INF-inducible guanosine triphosphate hydrolases (GTPases) superfamily and promotes tumorigenesis and cancer progression. It also plays an important role during host defense, which can affect autoimmunity, cancer-related immune response, and infection [28]. CD8A, an integral membrane glycoprotein, is crucial for differential immune responses to internal and external stimuli. It has served as an immune microenvironment biomarker in 14 different types of solid cancers, including SKCM, to identify patients that might benefit from anti-immunotherapies like anti-PD-1/PD-L1 and CAR-T treatment [29]. More research is warranted to examine the functions of the KIAA0040 and SAMHD1 genes in SKCM. Here, CD8A, GBP5, KIAA0040, and SAMHD1 expression were strongly correlated with DNA MET. As a result, drugs targeting the methylation process, such as azacitidine and 5-Aza-2-deoxycytidine, might help mitigate the effects of abnormal CD8A, GBP5, KIAA0040, and SAMHD1 levels, thus inhibiting progression of SKCM [30, 31].

In conclusion, this comprehensive analysis of epigenomic and genomic regulation of gene expression has revealed novel links between different transcriptional regulators in SKCM. Our findings might help identify novel immune-related molecular subtypes as well as new pathogenic mechanisms and clinical therapy targets in SKCM.

## MATERIALS AND METHODS

### DNA copy number, DNA MET, and mRNA expression profiles

The SKCM dataset was downloaded from TCGA. DNA copy number, DNA MET, mRNA expression patterns, and mutation data for SKCM samples were extracted through the official TCGA data portal. A total of 466 samples for which matched DNA copy number, DNA MET, and mRNA expression pattern data were available were included in this study (Supplementary Table 1). Gene expression profiles were normalized using quantile normalization and log2 transformation, and were later aggregated according to official HUGO symbols. Each expression profile was then normalized to mean expression of the probe gene in normal tissue so that it represented the fold change in cancer tissue relative to normal tissue. The circular binary segmentation algorithm from the R package “DNAcopy” library was then used to map genetic DNA copy numbers to CNV data in every sample according to the segmented CNV data [32]. For DNA MET patterns, probe β-values were filtered to remove probes located on sex chromosomes. Then, probes located in CpG island-associated regions were mapped to related genes, including Shelf, CpG islands, Shore regions, first-exon regions, differentially methylated regions, 5’-UTRs, and gene promoter regions containing 2500 upstream bases from TSS. Probes with >30% missing values across samples were removed from each processed profile.

The R package “liftOver” library was used to align probe genomic coordinates to the hg38 human reference genome in each dataset. Probes were then matched to corresponding probes in the mRNA expression profile data. In addition, cancer-specific alterations were identified by subtracting the average intensity of the probe in normal tissue. After probes with >50% missing values and those located on sex chromosomes had been removed, data were analyzed using the sk-nearest neighbor algorithm. Pairwise Pearson’s correlation coefficients were then calculated for each gene within the paired profiling patterns of CNV and expression and MET and EXP. If multiple probes had been mapped to the same gene, the probe with the mean or lowest correlation coefficient was used as the representative pair-matched probe for MET and CNV profiles, respectively.

### Clustering analysis of genome patterns from different aspects

Stable sample clusters were identified through negative matrix factorization (NMF) clustering analysis according to the “brunet” method and 50 iterations using METcor and CNVcor genes. The cluster number k was set between 2 and 10, and the optimal cluster number was computed based on the monitored consensus map as well as cluster cophenetic correlations. In addition, average silhouette width was determined for the consensus membership matrix using the R package “NMF.” For each member, the smallest cluster number was set to be 10. The “iCluster” R package was used to perform comprehensive clustering analyses of DNA CNV, MET, and mRNA expression profiles using default parameters and 20 iterations.

## Supplementary Material

Supplementary Figures

Supplementary Table 1

Supplementary Table 2

Supplementary Table 3

Supplementary Table 4

Supplementary Table 5

Supplementary Table 6

Supplementary Table 7

Supplementary Table 8

Supplementary Table 9

Supplementary Table 10

Supplementary Table 11

Supplementary Table 12

Supplementary Table 13
