# Supplementary material for: Epigenomic and genomic analysis of transcriptome modulation in skin cutaneous melanoma
Source: Aging (Albany NY). 2020 Jul 7;12(13):12703–25. doi: 10.18632/aging.103115 (PMC7377867; doi:10.18632/aging.103115)
Supplement: Supplementary Figures [file aging-12-103115-s008..pdf]

SUPPLEMENTARY FIGURES

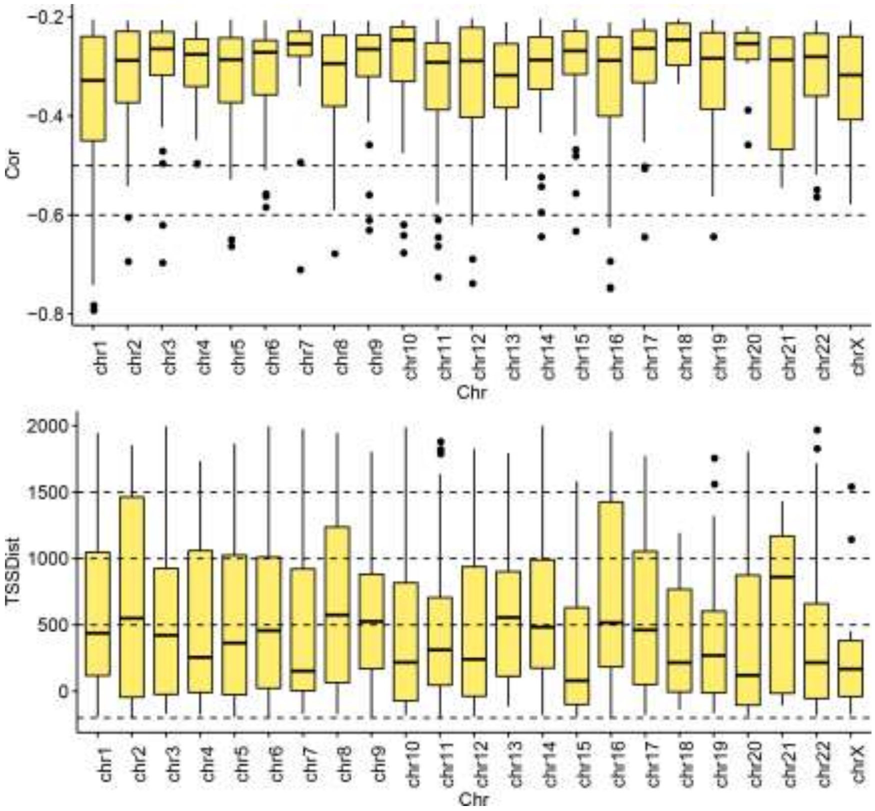

Supplementary Figure 1. The METCor genes' distribution on the genome.

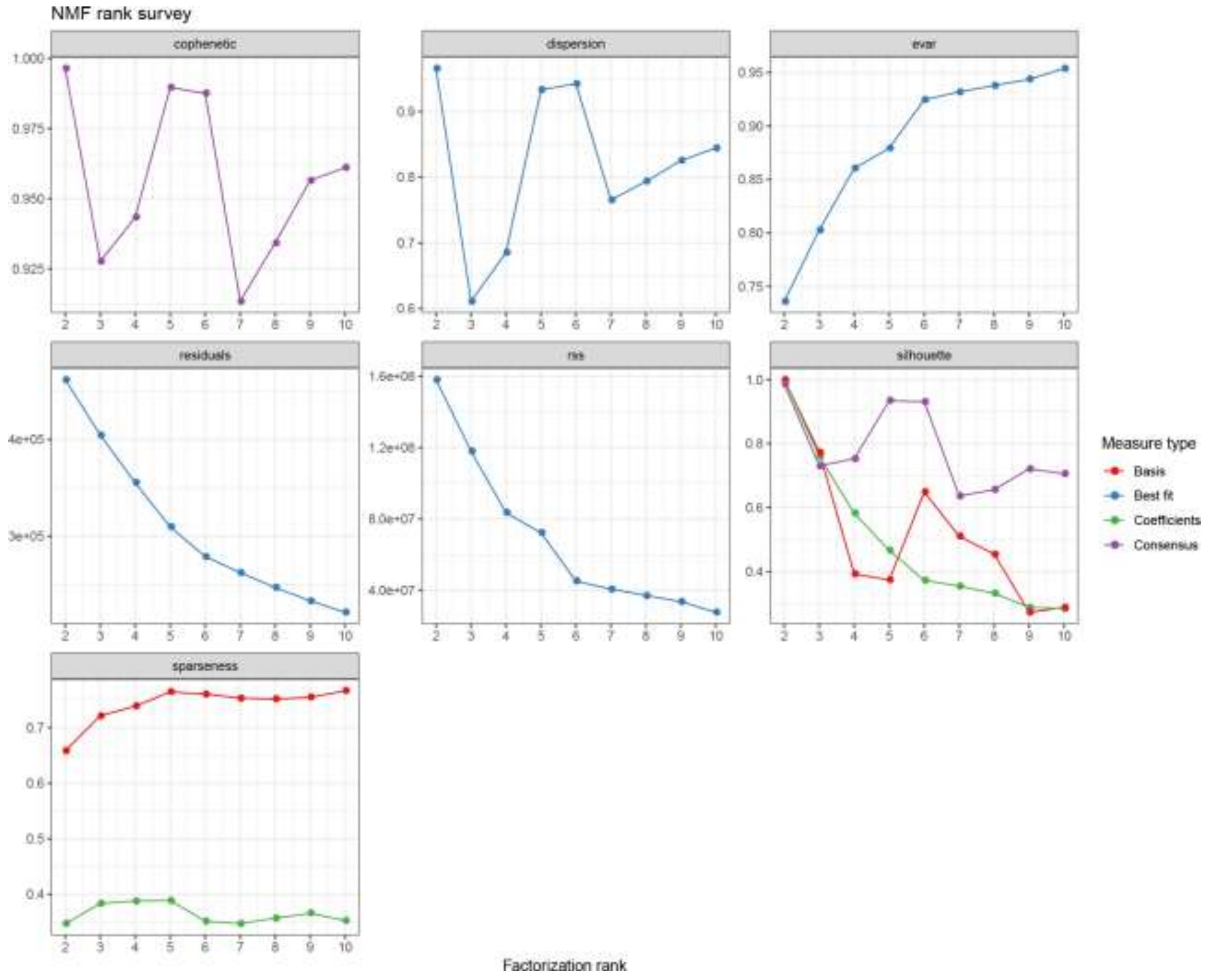

Supplementary Figure 2.NMF rank survey for CNVcor gene set. NMF: non-negative matrix factorization.

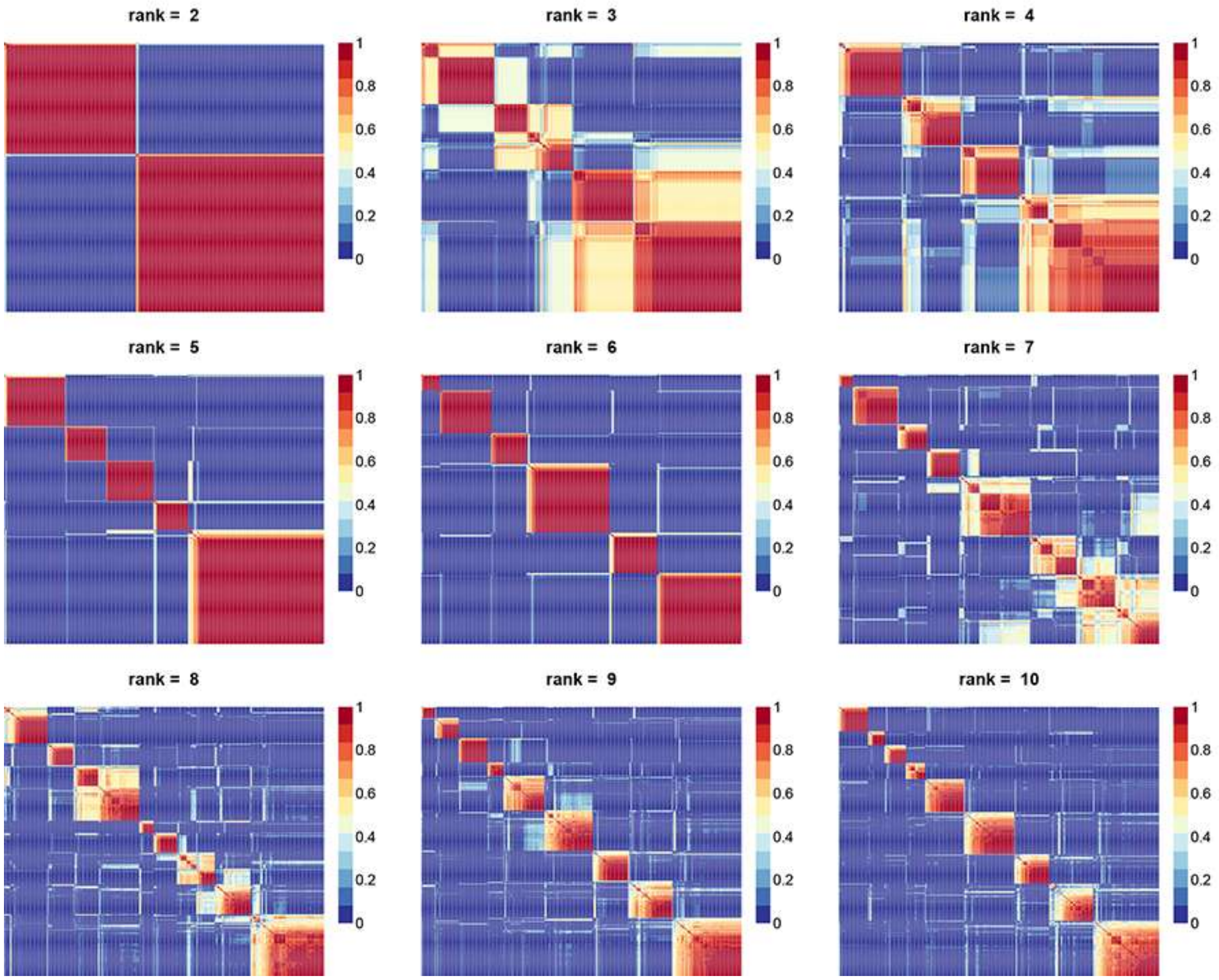

Supplementary Figure 3. NMF clustering analysis for CNVcor gene set. NMF: non-negative matrix factorization.

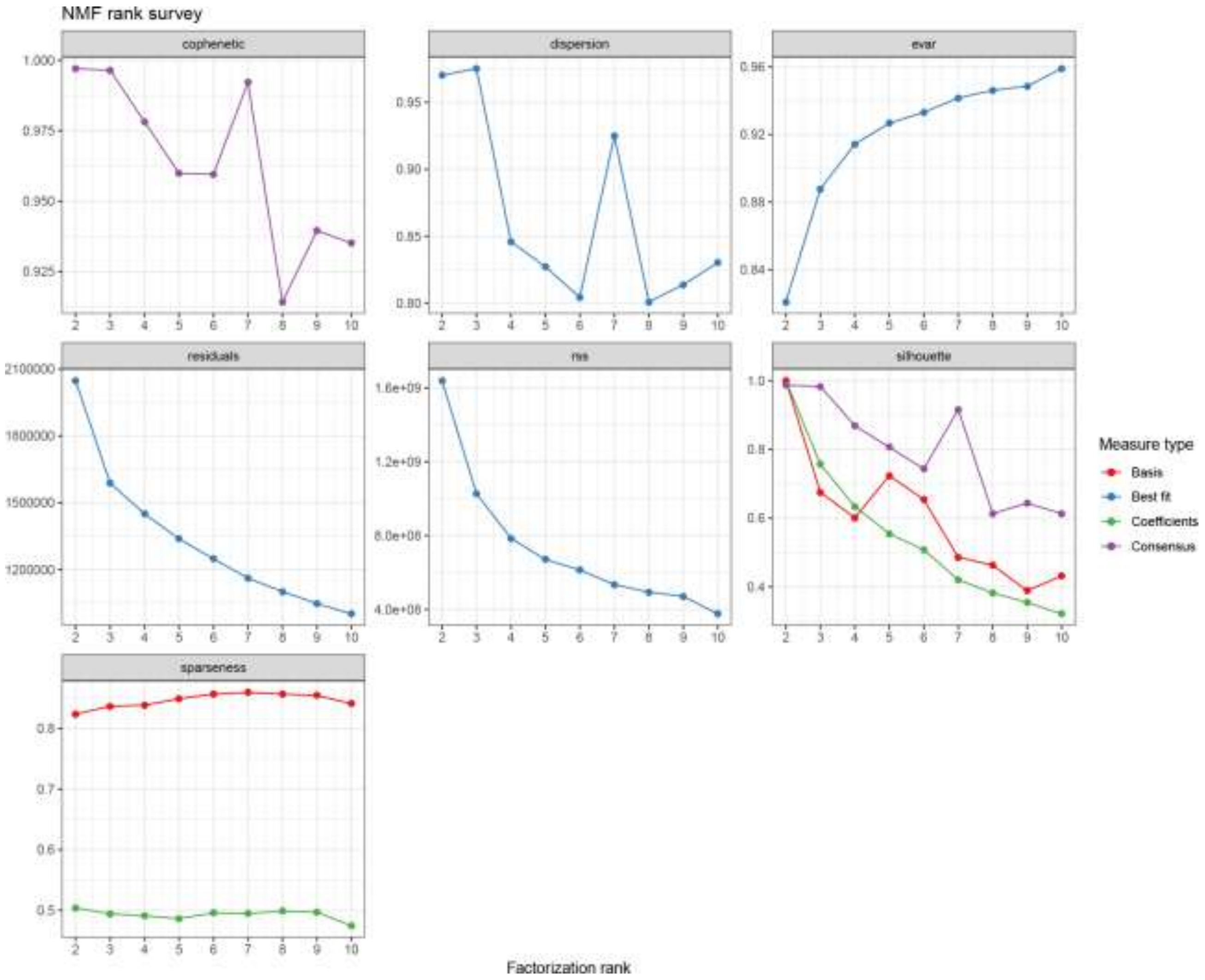

Supplementary Figure 4. NMF rank survey for METcor gene set. NMF: non-negative matrix factorization.

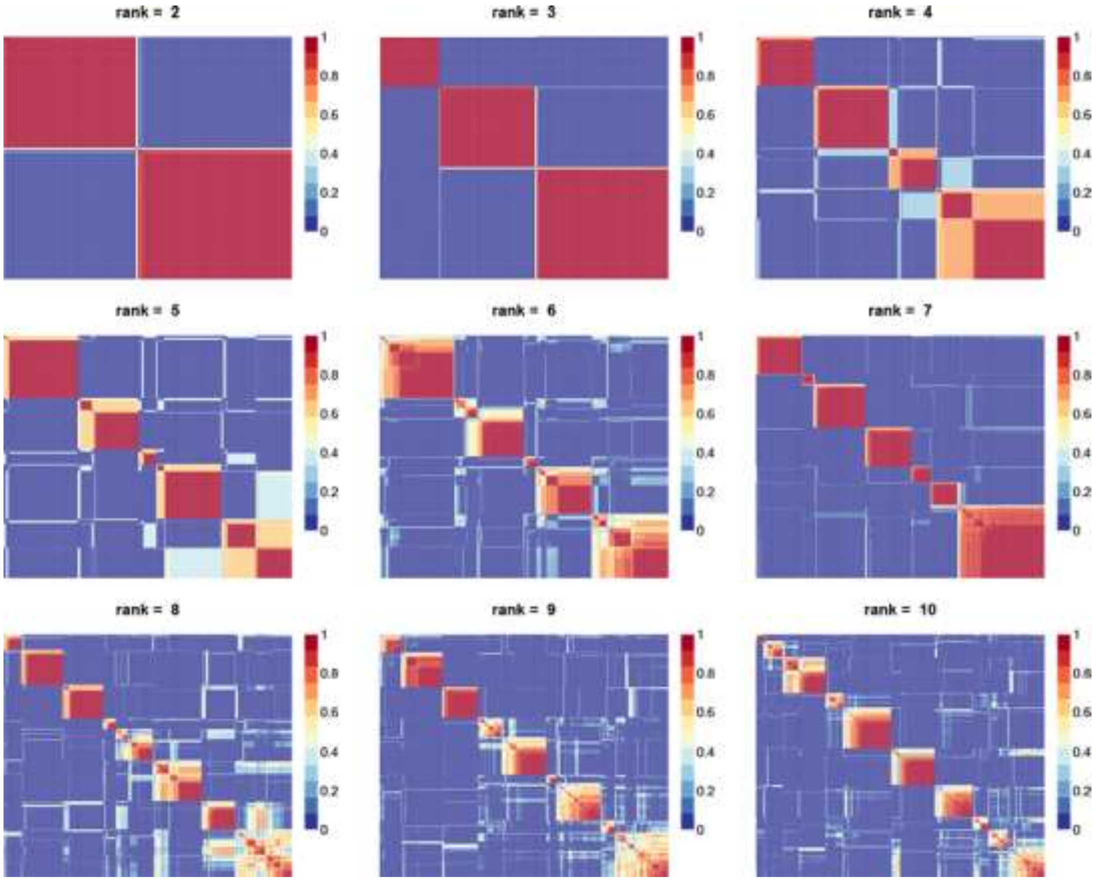

Supplementary Figure 5. NMF clustering analysis for METcor gene set. NMF: non-negative matrix factorization.

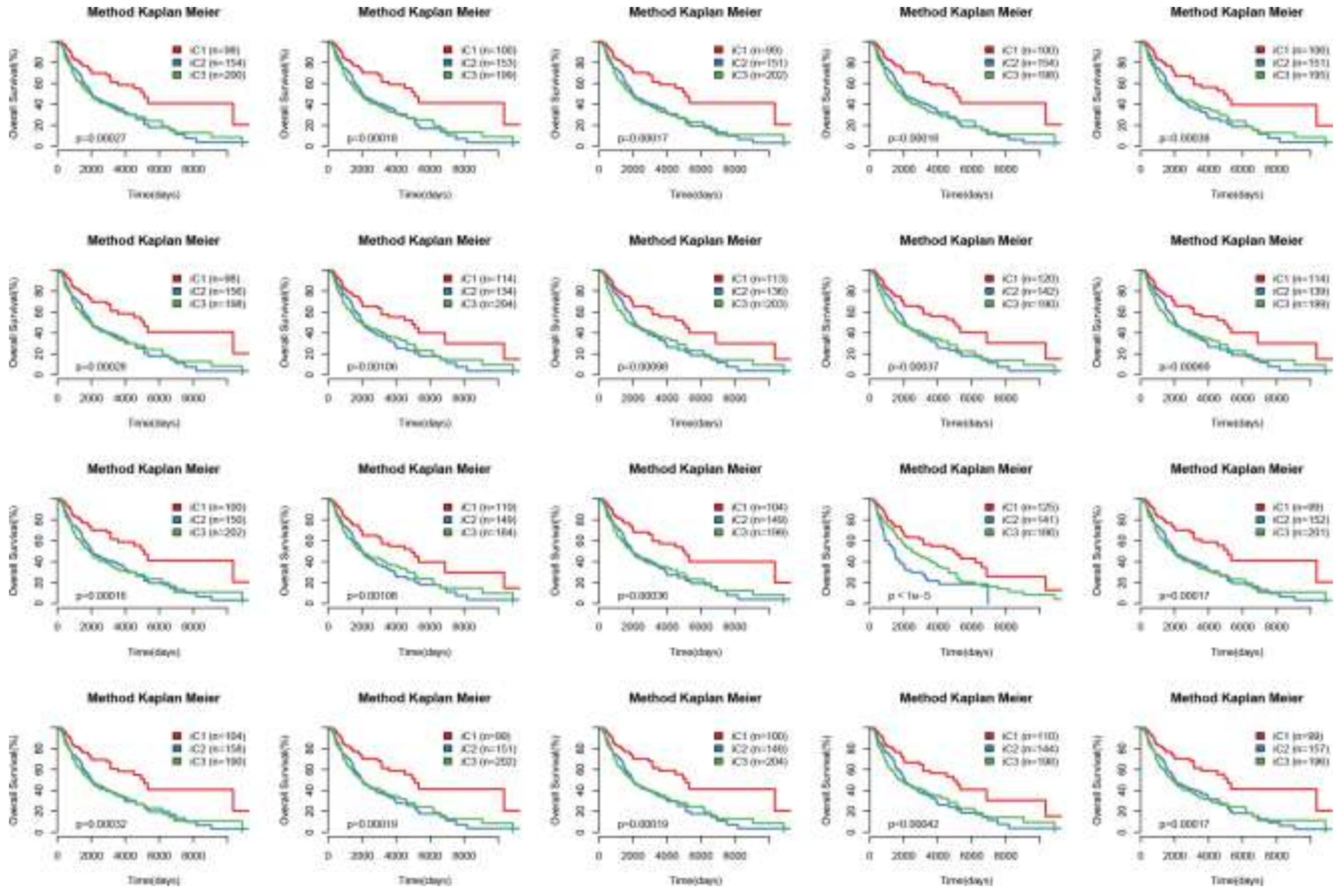

Supplementary Figure 6. iCluster multi-omics clustering result (K = 2, iC1-3).

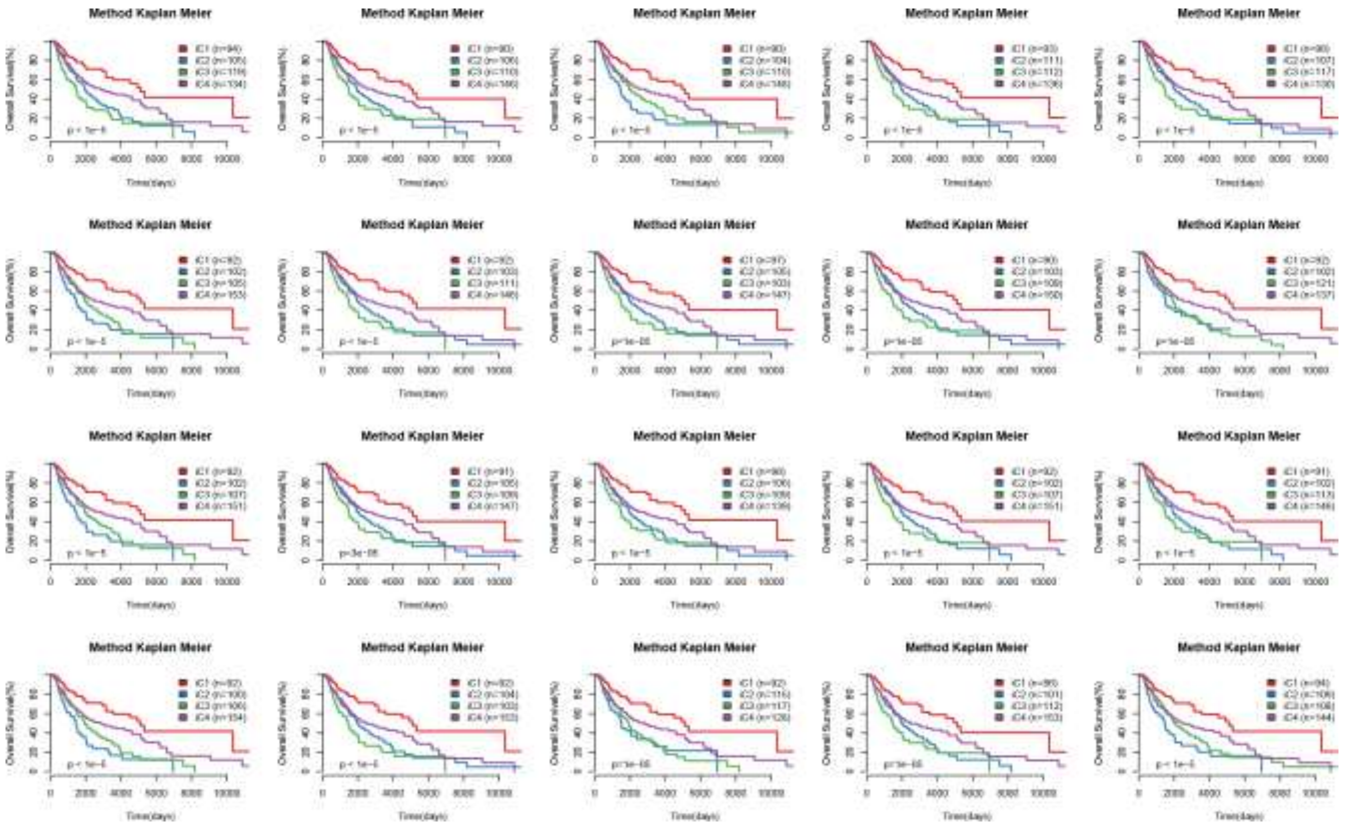

Supplementary Figure 7. iCluster multi-omics clustering result (K = 3, iC1-4).

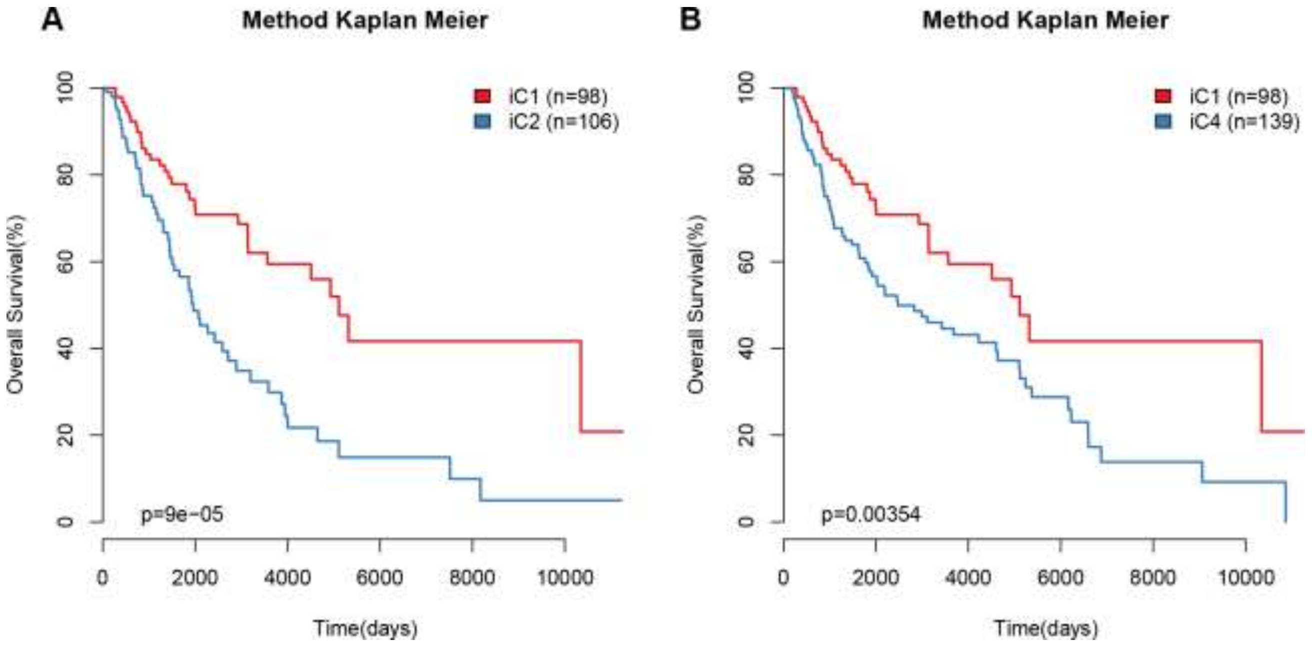

Supplementary Figure 8. Differences in prognosis between iC1 and iC2 / iC1 and iC4.

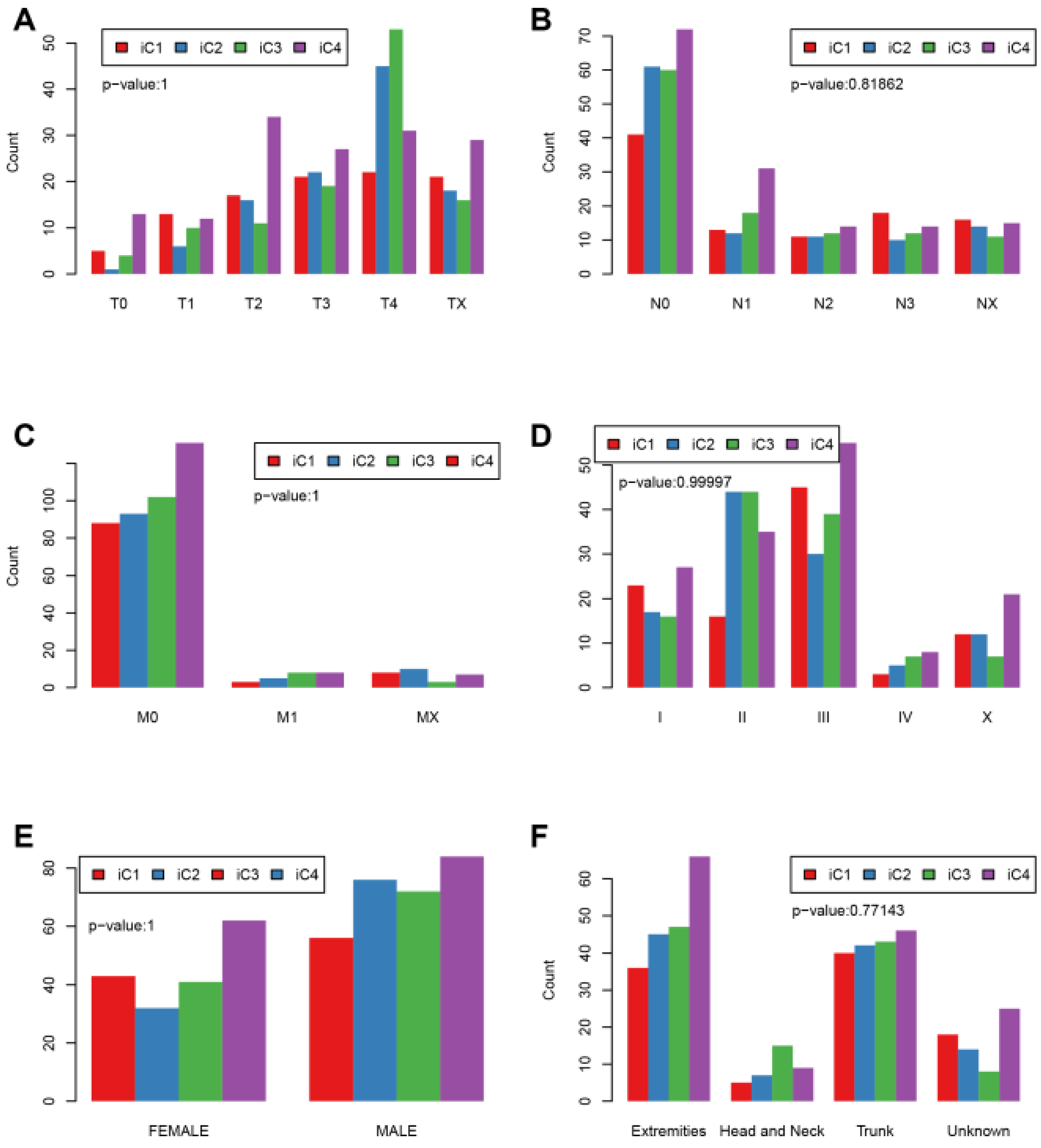

Supplementary Figure 9. Clinical characteristics (TNM, Stage, Gender, Primary site) differences between iC1-4.

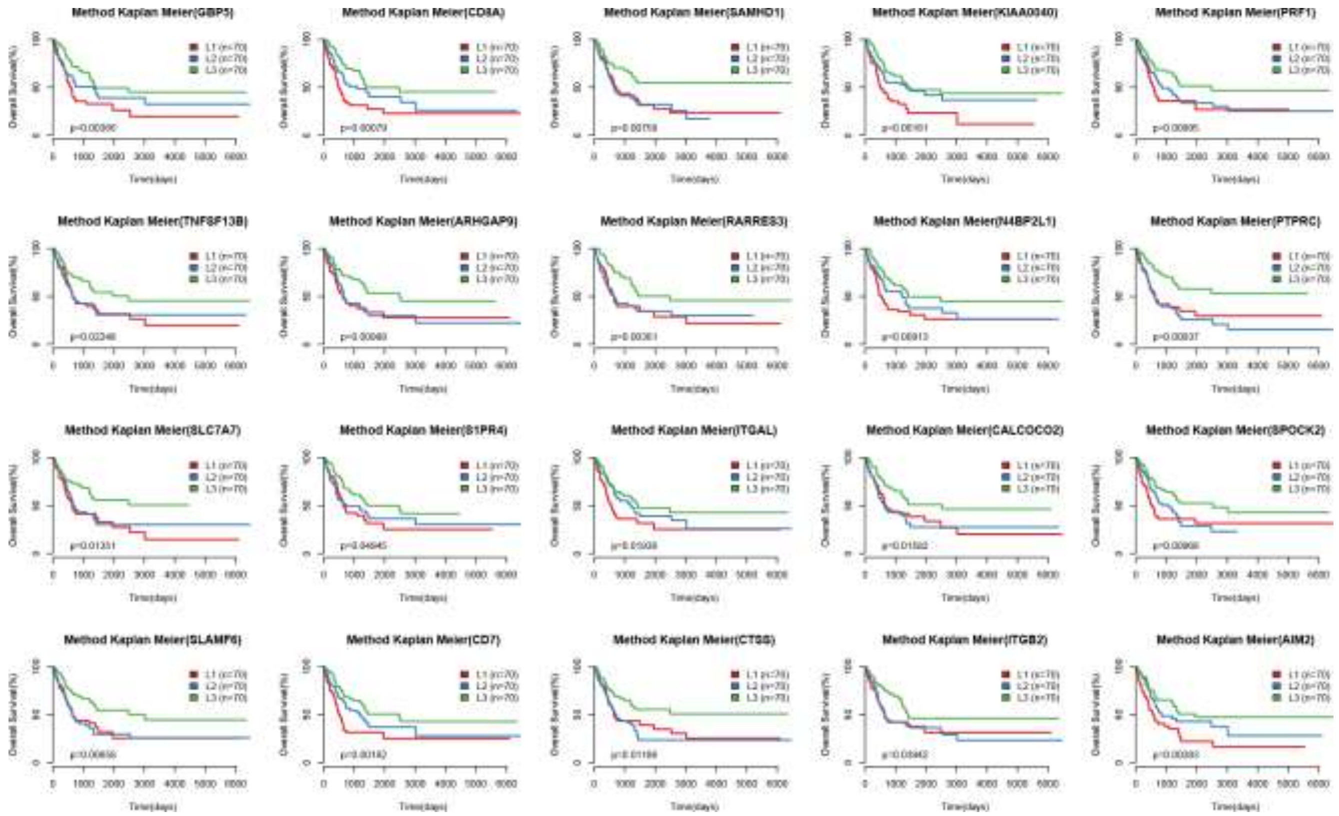

Supplementary Figure 10. Survival curves for the top 20 differential genes in the GEO validation data.
